# Crystal Initiation Structures in Developing Enamel: Possible Implications for Caries Dissolution of Enamel Crystals

**DOI:** 10.3389/fphys.2017.00405

**Published:** 2017-06-16

**Authors:** Colin Robinson, Simon D. Connell

**Affiliations:** ^1^School of Dentistry, University of LeedsLeeds, United Kingdom; ^2^Molecular and Nanoscale Physics Group, School of Physics and Astronomy, University of LeedsLeeds, United Kingdom

**Keywords:** enamel, crystals, initiation, assembly, caries

## Abstract

Investigations of developing enamel crystals using Atomic and Chemical Force Microscopy (AFM, CFM) have revealed a subunit structure. Subunits were seen in height images as collinear swellings about 30 nM in diameter on crystal surfaces. In friction mode they were visible as positive regions. These were similar in size (30–50 nM) to collinear spherical structures, presumably mineral matrix complexes, seen in developing enamel using a freeze fracturing/freeze etching procedure. More detailed AFM studies on mature enamel suggested that the 30–50 nM structures were composed of smaller units, ~10–15 nM in diameter. These were clustered in hexagonal or perhaps a spiral arrangement. It was suggested that these could be the imprints of initiation sites for mineral precipitation. The investigation aimed at examining original freeze etched images at high resolution to see if the smaller subunits observed using AFM in mature enamel were also present in developing enamel i.e., before loss of the organic matrix. The method used was freeze etching. Briefly samples of developing rat enamel were rapidly frozen, fractured under vacuum, and ice sublimed from the fractured surface. The fractured surface was shadowed with platinum or gold and the metal replica subjected to high resolution TEM. For AFM studies high-resolution tapping mode imaging of human mature enamel sections was performed in air under ambient conditions at a point midway between the cusp and the cervical margin. Both AFM and freeze etch studies showed structures 30–50 nM in diameter. AFM indicated that these may be clusters of somewhat smaller structures ~10–15 nM maybe hexagonally or spirally arranged. High resolution freeze etching images of very early enamel showed ~30–50 nM spherical structures in a disordered arrangement. No smaller units at 10–15 nM were clearly seen. However, when linear arrangements of 30–50 nM units were visible the picture was more complex but also smaller units including ~10–15 nM units could be observed.

**Conclusions:** Structures ~10–15 nM in diameter were detected in developing enamel. While the appearance was complex, these were most evident when the 30–5 nM structures were in linear arrays. Formation of linear arrays of subunits may be associated with the development of mineral initiation sites and attendant processing of matrix proteins.

## Introduction

Enamel comprises highly ordered crystals of substituted hydroxyapatite. These are of regular size and shape, densely packed with their long c-axes parallel and arranged in bundles, the enamel prisms. The precise mechanism of initiation and growth of these crystals is unclear.

Early transmission electron microscope (TEM) data suggested that crystals formed immediately outside of the ameloblast membrane, immediately after matrix secretion, appearing as thin needles or plates (Leblond and Warshawsky, 1979). However, to avoid the TEM preparation processes of dehydration, fixation and embedding in hydrophobic media which could induce premature precipitation and crystallization, early enamel was viewed using freeze etching which examines fractured surfaces of frozen unfixed tissue (Robinson et al., 1981). This revealed ~30 nm globular structures arranged both randomly and in linear arrays. Crystals only became visible during maturation after loss of matrix protein. The globules therefore are most likely complexes of amorphous mineral stabilized by protein. The dimensions and arrangement of these globules suggested that they are forerunners of the crystals seen in maturing enamel and delineate both the size, shape, and disposition of the crystals in mature tissue (Robinson et al., 1981). Subsequent investigations have supported the presence of amorphous mineral in early enamel (Aoba and Moreno, 1990; Rey et al., 1991; Diekwisch et al., 1995) which may also explain the very diffuse X ray diffraction patterns reported for early enamel (Nylen et al., 1963).

Globular crystal precursors were also supported by atomic and chemical force microscopy (AFM, CFM) of maturation stage enamel crystals. AFM revealed contiguous regular 30–50 nM globular swellings along maturation stage enamel crystals, redolent of the globules shown by freeze etching but which had subsequently fused and crystallized (Kirkham et al., 2001; Robinson et al., 2004) ultimately giving rise to the regular repeating charge domains on maturing crystals reported by Kirkham et al. (2000).

In addition, however, later high resolution AFM indicated that the 30–50 nM globular structures comprised smaller ~15 nM subunits arranged in roughly hexagonal or possibly spiral patterns (Robinson et al., 2004, 2006). Since these may represent imprints of original crystal initiation structures, earlier freeze etched data was re-examined at high resolution for their presence. High resolution freeze etched images, did reveal ~15 nM substructures within the original globules. *These appeared more obviously as the globules formed linear arrays, possibly reflecting matrix processing associated with transition from amorphous mineral to crystals*.

## Materials and methods

Freeze etching of early enamel was reported by Robinson et al. (1981). Briefly, early enamel was carefully frozen in liquid nitrogen (−198°C) and fractured under vacuum using a histological knife. The knife was then repositioned over the fractured surface and its temperature lowered to sublime ice from the tissue on to the knife blade. This left a fractured tissue surface unencumbered by ice. The fractured frozen surface was then shadowed, under vacuum, with gold or aluminum. Tissue was dissolved away and the metal replica examined using TEM.

AFM was carried out as described previously (Kirkham et al., 2001; Robinson et al., 2004) using a Nanoscope III AFM (Digital Instruments) equipped with a 16E16-μm scanner and 25 μm silicon nitride cantilevers. Images were obtained in oscillating mode at 0.2 Hz below resonance with drive amplitudes in the range 300–950 mV. Measurements of crystal width and height were made using the software provided.

## Results and discussion

**Figure 1:**
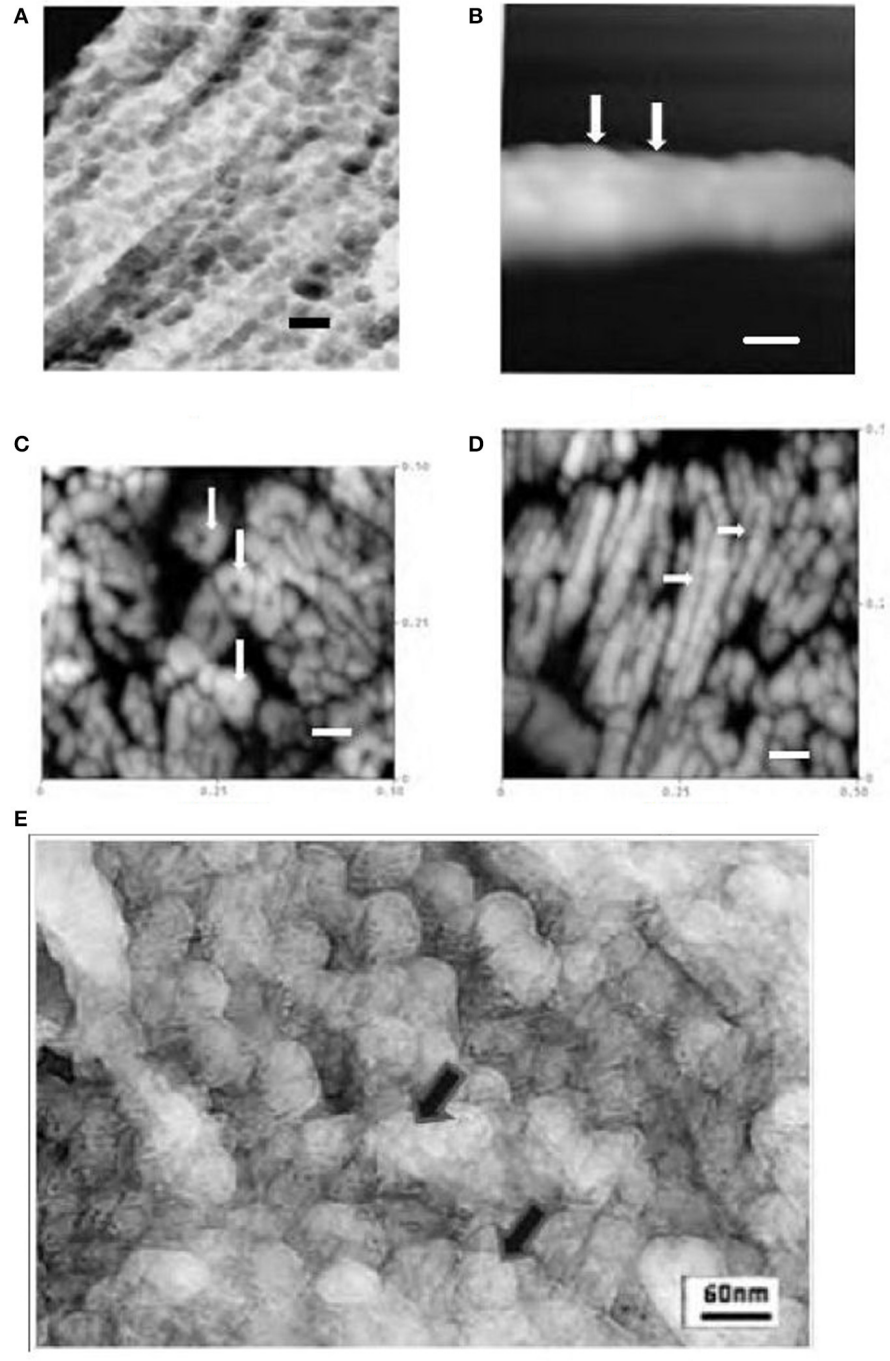
**(A)** TEM freeze etched enamel showing repeated globular structures ~30–50 nM dia in linear arrays (rat incisor) (Robinson et al., 1981) (bar = 60 nM). **(B)** AFM tapping mode in air of *maturation stage* enamel crystal showing repeated contiguous globular subunits ~30 nM diameter (rat incisor, tapping mode in air) (Kirkham et al., 2000; Robinson et al., 2004) arrows (bar = 30 nM).**(C)**. AFM image of polished section of mature human enamel. Cross sections of enamel crystals are visible showing ~15 nM subunits in roughly hexagonal clusters (tapping mode in air) (Robinson et al., 2004) arrows (bar = 60 nM). **(D)** AFM image of polished section of mature human enamel. Longitudinal sections of enamel crystals are visible showing 15nm subunits, (human, tapping mode in air) longitudinal interface between subunits can be seen arrows (Robinson et al., 2004) (bar = 60 nM). **(E)** High resolution TEM image of freeze etched rat incisor secretory enamel showing 30–50 nM globules but comprising smaller ~15 nM subunits, arrows.

As previously reported, the data shown illustrates the presence of 30–50 nM diameter globules in secretory enamel, arranged randomly or in linear arrays (Robinson et al., 1981), Figure 1A. That these represent crystal forerunners was supported by high resolution AFM height images of deproteinated maturation enamel crystals (Kirkham et al., 2001). AFM images revealed contiguous 30–50 nM diameter swellings along crystal surfaces presumably representing mineralized replacements of original matrix -mineral structures, Figure 1B.

However, high resolution AFM images of mature enamel also revealed previously unreported 15 nM substructures within the ~30 nM globules arranged in roughly hexagonal or perhaps spiral patterns (Robinson et al., 2004, 2006), Figures 1C,D. These most likely represent original mineral initiation structures comprising amorphous mineral stabilized by matrix proteins. While the original freeze etching investigation reported 30–50 nM globules, it did not refer to any smaller structures, the images had not, however, been examined at high resolution. When this was carried out smaller globules 15 nM in size were in fact visible, Figure 1E. Although it was not possible to discern exactly how these were arranged they are clearly forerunners of the fully mineralized 15 nM subunits seen in mature enamel crystals.

Approximately 15 nM units of enamel structure have also been reported using other techniques. Diekwisch (1998) reported polygonal, possibly mineral particles at about ~15 nM adjacent to secretory ameloblasts and more recently Beniash et al. (2009) using TEM, showed linear arrays of spherical particles each measuring about 15 nM. This study also used electron diffraction, FITR XPEEM and demonstrated that amorphous mineral was present.

These 15 nM structures may be amorphous mineral *per-se* but are more likely to be mineral matrix complexes. That they appeared more clearly when 30 nM globules lined up suggests that matrix processing may be involved in alignment and mineral precipitation see below (Fang et al., 2011). It is proposed that the ~15 nM subunits represent mineral initiation sites where mineral nuclei precipitate and subsequently fuse both into long chains and laterally into wider 30–50 nM structures before transforming into hydroxyapatite.

**Figure 2:**
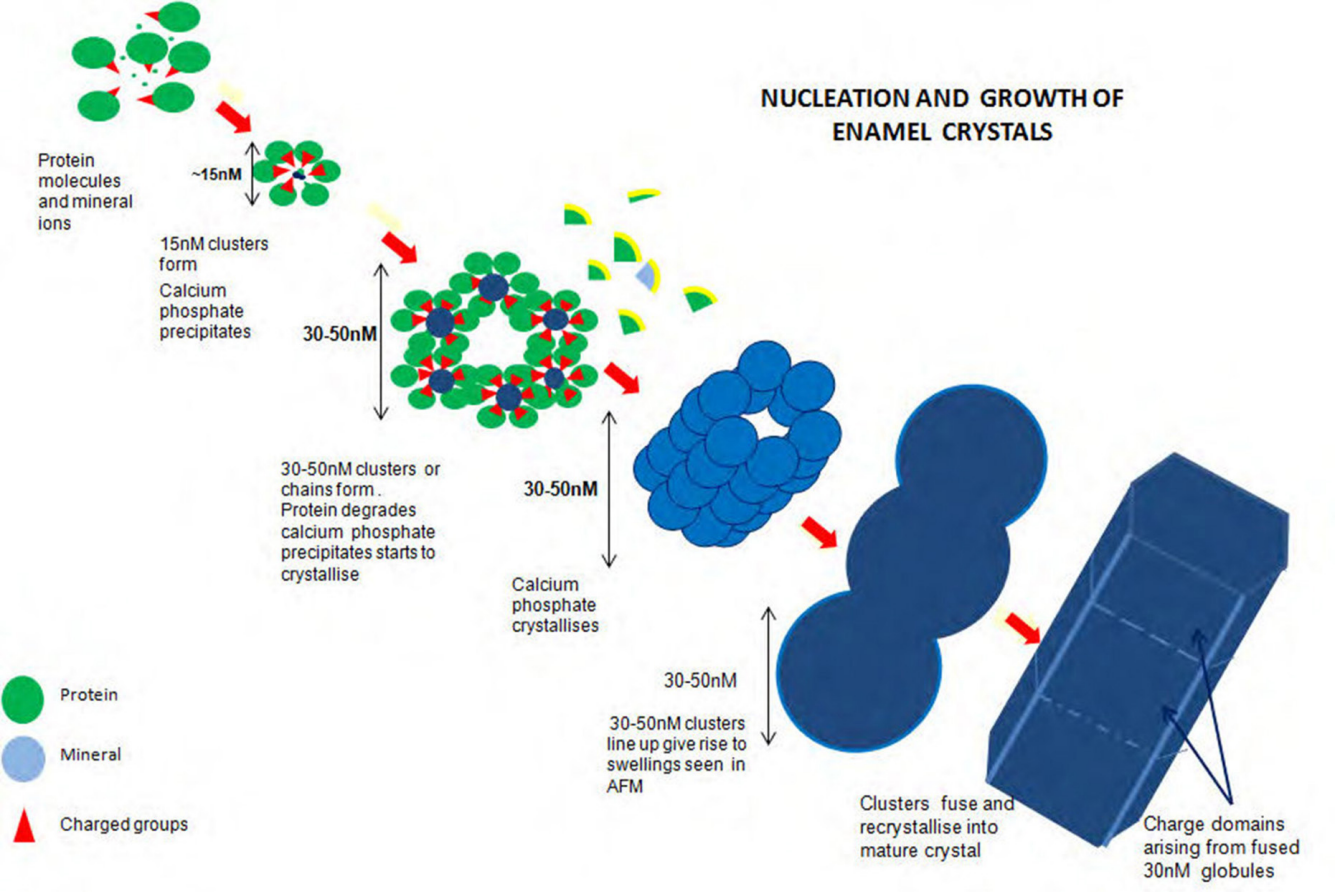
Diagram of proposed formation of enamel crystals from ~15 nM protein mineral complexes to the fully mature crystal. Fifteen nM structures form comprising mineral ions stabilized by matrix protein. These assemble, either as linear strings which fuse laterally to produce long chains of roughly hexagonal clusters or the hexagonal clusters themselves form and assemble lengthwise to produce long chains of roughly hexagonal clusters. Removal of matrix at some point results in mineral precipitation and transformation to apatite and the clusters fuse to become chains of globular structures ~30 nM diameter. Recrystallization results in the mature enamel crystal with crystalline or chemical discontinuities at the fusion interfaces.

Figure 2 illustrates the proposed formation of enamel crystals from~15 nM protein mineral complexes to the fully mature crystal. 15 nM structures form comprising mineral ions stabilized by matrix protein. These assemble, either as linear strings which fuse laterally to produce long chains of roughly hexagonal clusters or the hexagonal clusters themselves form and assemble lengthwise to produce long chains of roughly hexagonal clusters. Removal of matrix at some point results in mineral precipitation and transformation to apatite and the clusters fuse to become chains of globular structures ~30 nM diameter. Recrystallisation results in the mature enamel crystal with crystalline or chemical discontinuities at the fusion interfaces.

It is not yet known precisely how the amorphous material is initiated and temporarily stabilized. Initiation may occur within the 15 nM subunits if ionic peptide side chains, for example, the C terminal peptide of amelogenin and/or its phosphate group are turned inward and the subunits held together by hydrophobic interaction (Figure 2).

The rapid loss of the hydrophilic C terminal of amelogenin and loss of the phosphate group have been implicated in the transformation from stabilized amorphous mineral to crystalline phase (see Kwak et al., 2011; Khan et al., 2012). Loss of phosphate may be related to the presence of phosphatase activity reported by Robinson et al. (1990) and Moe et al. (1996). *In vitro* investigations using amelogenin (Fang et al., 2011) have indicated the capacity of amelogenin not only to stabilize amorphous calcium phosphate but also to foster the development of long apatite crystal bundles by oligomeric organization into chains. This does not, however, preclude the effect of further protein processing or a role for other enamel proteins such as enamelin and ameloblastin. It should also be borne in mind that the decrease in the high concentrations of carbonate and magnesium present in early enamel could effect a transformation from amorphous to crystalline phase (Hiller et al., 1975; Aoba and Moreno, 1990; Rey et al., 1991).

The significance of clustering of 15 nM initiation sites is significant from a number of points of view. From the viewpoint of enamel structure, clustering into 30–50 nM units delineates the ultimate crystal width and thickness thus outlining tissue volume to be occupied by crystals. This is important since the matrix is ultimately removed.

This also has implications for enamel caries since the fused interface between these units would lead to increased acid solubility due to crystalline discontinuity (see Robinson et al., 2004). Chemical discontinuity may also occur as high concentrations of carbonate and magnesium may be moved to these interfaces during recrystallization associated with crystal growth. Lateral fusion would lead to a discontinuity along the length of the crystal at its center, while longitudinal fusion would lead to lateral discontinuities perpendicular to the central line. These are the sites at which enamel crystals are known to dissolve preferentially during carious attack (Johnson, 1967; Yanagisawa and Miake, 2003).

## Ethics statement

Wistar rats were maintained and killed according to local and national animal regulations. University of Leeds Dental School Animal Committee. Human extracted teeth were obtained from the tissue bank at Leeds dental School. Teeth were obtained according to national and local guidelines permission obtained at source.

## Author contributions

CR designed and set up both investigations and was largely responsible for freeze etch studies. SC carried out and advised upon AFM and CFM investigations. Both authors interpreted data and discussed implications, both were involved in writing.

### Conflict of interest statement

The authors declare that the research was conducted in the absence of any commercial or financial relationships that could be construed as a potential conflict of interest.

## References

[B1] Aoba T. Moreno E. C. (1990). Changes in the nature and composition of enamel mineral during porcine amelogenesis. Calcif. Tissue Int. 47, 356–364. 10.1007/BF02555887 1963381

[B2] Beniash E. Metzler R. A. Lam R. S. Gilbert P. (2009). Transient amorphous calcium phosphate in forming enamel. J. Struct. Biol. 166, 133–143. 10.1016/j.jsb.2009.02.001 19217943 PMC2731811

[B3] Diekwisch T. (1998). Subunit compartments of secretory stage enamel matrix. Connect. Tissue Res. 38, 101–111. 10.3109/03008209809017026 11063019

[B4] Diekwisch T. G. H. Berman B. J. Gentner S. Slavkin H. C. (1995). Initial enamel crystals are not spatially associated with mineralized dentine. Cell Tissue Res. 279, 149–167. 7895256 10.1007/BF00300701

[B5] Fang P. Conway J. F. Margolis H. C. Simmer J. P. Beniash E. (2011). Hierarchical self-assembly of amelogenin and the regulation of biomineralization at the nanoscale. Proc. Nat. Acad. Sci. U.S.A. 34, 14097–14102. 10.1073/pnas.1106228108 PMC3161614 21825148

[B6] Hiller C. R. Robinson C. Weatherell J. A. (1975). Variations in the composition of developing rat incisor enamel. Calcif. Tiss. Res. 18, 1–12. 10.1007/BF02546222 1148889

[B7] Johnson N. W. (1967). Some aspects of the ultrastructure of early human enamel caries seen with the electron microscope. Archs oral Biol. 12, l505–1521. 10.1016/0003-9969(67)90186-0 5237335

[B8] Khan F. Li W. Habelitz S. (2012). Biophysical characterization of synthetic amelogenin C-terminal peptides. Eur. J. Oral Sci. 120, 113–122. 10.1111/j.1600-0722.2012.00941.x 22409217 PMC3306135

[B9] Kirkham J. Brookes S. J. Zhang J. Wood S. R. Shore R. C. Smith D. A.. (2001). Effect of experimental fluorosis on the surface topography of developing enamel crystals. Caries Res. 35, 50–56. 10.1159/000047431 11125197

[B10] Kirkham J. Brookes S. J. Zhang J. Wood S. R. Shore R. C. Smith D. A.. (2000). Evidence for charge domains on developing enamel crystal surfaces. J. Dent. Res. 79, 1943–1947. 10.1177/00220345000790120401 11201043

[B11] Kwak S. Y. Green S. Wiedemann-Bidlack F. B. Beniash E. Yamakoshi Y. C. (2011). Regulation of calcium phosphate formation by amelogenins under physiological conditions. Eur. J. Oral Sci. 119, 103–111. 10.1111/j.1600-0722.2011.00911.x 22243235 PMC3448280

[B12] Leblond C. P. Warshawsky H. (1979). Dynamics of enamel formation in the rat incisor tooth. J. Dent. Res. 58, 950–975. 10.1177/00220345790580024901 283137

[B13] Moe D. Kirkeby S. Salling E. (1996). Biochemical characterisation of alkaline phosphatase from partly mineralized bovine enamel. J. Biol. Buccale 14, 249–253. 3468106

[B14] Nylen M. U. Eanes E. D. Omnell K. A. (1963). Crystal growth in rat enamel. J. Cell Biol. 18, 109–123. 10.1083/jcb.18.1.109 13939321 PMC2106281

[B15] Rey C. Shimizu M. Collins B. Glimcher M. (1991). Resolution-enhanced Fourier transform infrared spectroscopy study of the environment of phosphate ion in the early deposits of a solid phase of calcium phosphate in bone and enamel and their evolution with age. 2. Investigations in the v3 PO4 domain. Calcif. Tissue Int. 49, 383–388. 10.1007/BF02555847 1818762

[B16] Robinson C. Connell S. Kirkham J. Shore R. C. Smith A. (2004). Dental enamel, a biological ceramic: regular substructures in enamel hydroxyapatite crystals revealed by atomic force microscopy. J. Mater. Chem. 14, 2242–2248. 10.1039/b401154f

[B17] Robinson C. Fuchs P. Weatherell J. A. (1981). The appearance of developing rat incisor enamel using a freeze fracturing technique. J. Cryst. Growth 53, 160–165. 10.1016/0022-0248(81)90062-2

[B18] Robinson C. Shore R. C. Kirkham J. Stonehouse N. J. (1990). Extracellular processing of enamel matrix proteins and the control of crystal growth. J. Biol. Buccale 18, 355–361. 1965651

[B19] Robinson C. Yamamoto K. Connell S. Kirkham J. Nakagaki H. Smith A. D. (2006). The effects of fluoride on the nanostructure and surface pK of enamel crystals. An atomic force microscopy study of human and rat enamel. Eur. J Oral Sci. 114, 1–10. 10.1111/j.1600-0722.2006.00275.x 16674669

[B20] Yanagisawa T. Miake Y. (2003). High-resolution electron microscopy of enamel crystal demineralization and remineralization in carious lesions. J. Electron. Microsc. 52, 605–613. 10.1093/jmicro/52.6.605 14756249

